# Ultrahigh-temperature melt printing of multi-principal element alloys

**DOI:** 10.1038/s41467-022-34471-7

**Published:** 2022-11-07

**Authors:** Xizheng Wang, Yunhao Zhao, Gang Chen, Xinpeng Zhao, Chuan Liu, Soumya Sridar, Luis Fernando Ladinos Pizano, Shuke Li, Alexandra H. Brozena, Miao Guo, Hanlei Zhang, Yuankang Wang, Wei Xiong, Liangbing Hu

**Affiliations:** 1 Department of Materials Science and Engineering, University of Maryland, College Park, Maryland 20742 USA; 2 Center for Materials Innovation, University of Maryland, College Park, Maryland, 20742, USA; 3 Department of Mechanical Engineering and Materials Science, University of Pittsburgh, Pittsburgh, PA 15261 USA; 4 Center for Hierarchical Materials Design, Northwestern University, Evanston, IL 60208 USA

**Keywords:** Design, synthesis and processing, Metals and alloys

## Abstract

Multi-principal element alloys (MPEA) demonstrate superior synergetic properties compared to single-element predominated traditional alloys. However, the rapid melting and uniform mixing of multi-elements for the fabrication of MPEA structural materials by metallic 3D printing is challenging as it is difficult to achieve both a high temperature and uniform temperature distribution in a sufficient heating source simultaneously. Herein, we report an ultrahigh-temperature melt printing method that can achieve rapid multi-elemental melting and uniform mixing for MPEA fabrication. In a typical fabrication process, multi-elemental metal powders are loaded into a high-temperature column zone that can be heated up to 3000 K via Joule heating, followed by melting on the order of milliseconds and mixing into homogenous alloys, which we attribute to the sufficiently uniform high-temperature heating zone. As proof-of-concept, we successfully fabricated single-phase bulk NiFeCrCo MPEA with uniform grain size. This ultrahigh-temperature rapid melt printing process provides excellent potential toward MPEA 3D printing.

## Abstract (web summary)

Multi-principal element alloy (MPEA) 3D printing is challenging due to the tradeoff between achieving high-temperature and sufficient heating zone. Here, the authors report an ultrahigh-temperature melt printing method that can achieve rapid melting and uniform elemental mixing for MPEA fabrication.

## Introduction

Multi-principal element alloys (MPEA) feature three or more principal elements present in significant amounts, unlike traditional alloys which consist of predominantly one element^1–5^. MPEAs display unique and extensively tunable properties due to their significantly expanded compositional design space^6–9^. Synergetic physical, chemical, and mechanical properties can be achieved by screening appropriate combinations of metals^6,10,11^. For example, CrCoNi-based MPEAs demonstrate excellent strength, ductility, corrosion and oxidation resistance, surpassing some of the best performing single-principal Ni-based superalloys and Fe-based stainless steels^8,12,13^. MPEAs composed of low density elements, such as Al, Mg, Be, and Ti, also have great potential in the automotive and aerospace industries due to their light weight^14–16^. However, the homogeneous mixing of multi-principal elements is challenging as it requires a powerful heating source to fully melt and homogeneously mix various dissimilar elements, which is particularly difficult considering that the melting temperatures may take place over a wide range.

Three-dimensional (3D) printing is an emerging process with a great potential to fabricate geometrically complex MPEA structural products with favorable properties and performances^6,8,17–19^. To achieve a high heating temperature for rapid multi-elemental melting/mixing during printing, focused high-energy sources (e.g., lasers, electron beam, electric arc) are commonly used to interact and melt metal powders into dense products^20–22^. The relatively rapid cooling rate of these methods can effectively prevent the formation of undesired intermetallic phases^23–25^. While the temperature of these heating sources is sufficiently high to melt a wide range of elements, these approaches can only achieve a small-sized melt zone (e.g., the laser beam diameter is usually only ~100 µm), which results in a highly uneven temperature distribution^26–28^. Due to the rapid solidification of the small melt pool and inhomogeneous temperature, insufficient thermal transport and diffusion of the elements occur, which causes chemical and microstructural inhomogeneity in the final MPEA products^29–31^. As a result, 3D printing MPEAs using a focused high-energy heating source typically requires the multi-elemental metals to be pre-alloyed, which adds extra processing costs^32–34^. As an alternative, direct liquid metal printing methods have been developed, in which the solid metals are melted by a printhead that is electrically pre-heated using copper coils^35,36^. However, the temperature of the copper heating coils is generally limited to <1000 K, constraining the range of metals that can be employed with this technique. Additionally, copper coils usually suffer from low radiative heating power due to low emissivity of copper (<0.05)^37^. As a result, there is a tradeoff in current MPEA printing techniques between sufficient high temperature for multi-elemental melting/mixing and an optimal melt pool with uniform temperature distribution for homogeneous elemental diffusion and the fabrication of MPEAs.

**Fig. 1:**
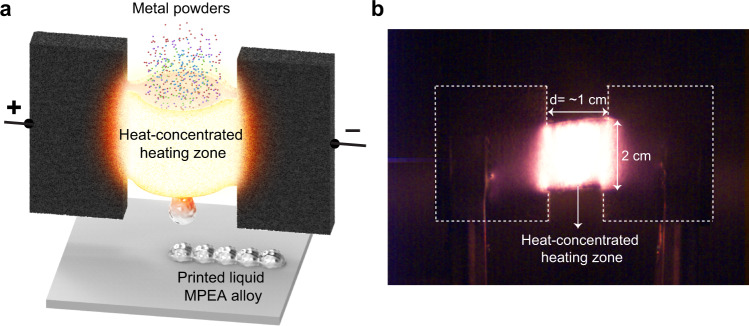
Ultrahigh-temperature melt printing platform for multi-elemental mixing to fabricate MPEAs. **a** Schematic of the ultrahigh-temperature, heat-concentrated platform for multi-elemental metal melt mixing towards the fabrication of MPEAs. Due to the higher electrical resistance of the heating zone, only this area of the carbon felt produces a high temperature under the application of a voltage due to Joule heating. Multi-elemental metal powders are loaded continuously into the heat-concentrated zone of which the temperature can be tuned to as high as 3000 K with a uniform temperature distribution. The metal powders are rapidly heated, melted on the order of milliseconds, and alloyed into uniform MPEAs. **b** Photograph of the ultrahigh-temperature, heat-concentrated platform for multi-elemental metal melt mixing.

In this work, we demonstrate an ultrahigh-temperature melt printing platform that has great potential as a heating source for the fabrication of MPEAs towards 3D printing. The heating platform is enabled by Joule heating a carbon felt substrate that is split down the middle to create a column with narrow wall thicknesses. This structure creates an area of higher electrical resistance that produces a heat-concentrated zone down the center of the column. In a typical fabrication process (Fig. 1), multi-elemental metal powders are loaded continuously into the heat-concentrated zone of which the temperature can be tuned to as high as 3000 K with a uniform temperature distribution. Compared with conventional focused high-energy beams (e.g., a 100 µm diameter laser spot size), our heating zone features a much larger area (~10 mm in diameter), which significantly promotes the uniform melting/mixing of multi-elemental metal powders. The metal powders are rapidly heated and melted through radiation and conduction on the order of milliseconds as they move down the heating zone. The bottom of the heating column is sealed with carbon felt until liquid alloy droplets are formed, which are then extruded from the heating zone by removing the sealed carbon felt, followed by cooling down with a rate of hundreds of K/s. Such rapid quenching is beneficial for achieving a homogenous multi-elemental chemical composition and microstructure with uniform grain size. Additionally, the rapid melting helps minimize the loss of volatile metallic elements during heating. As a proof-of-concept demonstration, we fabricated a single face-centered-cubic (FCC) phase NiFeCrCo MPEA featuring uniform elemental distribution. The high heating temperature and relatively large heating zone simultaneously enable rapid and uniform melting and subsequent cooling to produce a highly homogenous MPEA. In addition, compared with conventional alloys fabricated by arc melting, the materials fabricated by our ultrahigh-temperature melt printing platform feature smaller uniform grain size and less loss of volatile elements due to the rapid heating and cooling process. As a result, this heat-concentrated, high-temperature platform shows strong potential for the rapid fabrication of MPEA structural materials towards 3D printing.

## Results

### Temperature characterization of the heat-concentrated reaction zone

**Fig. 2:**
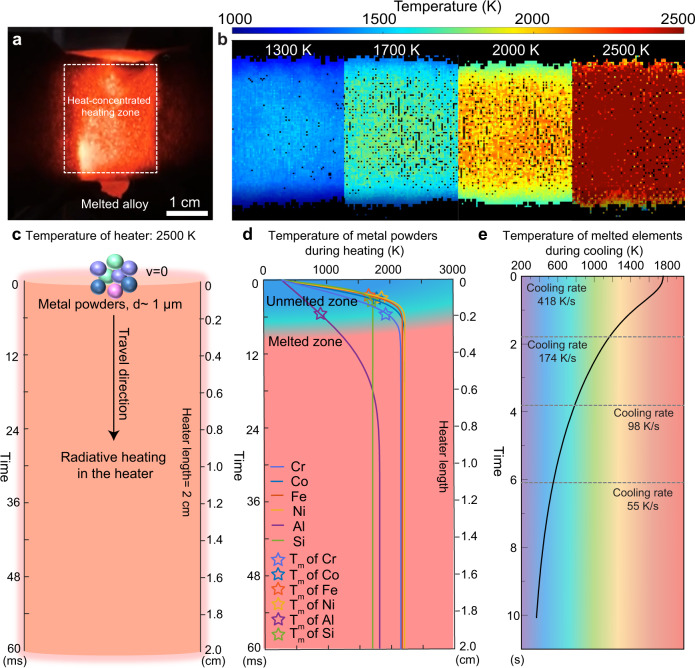
Temperature of the heat-concentrated zone and the heating and cooling process of the metal powders. **a** Photograph of the heat-concentrated heater extruding a melted bulk MPEA droplet. When applying a voltage, the heat is concentrated in the column region, emitting a bright light, while the edges of the carbon felt with lower electrical resistance remain dark. **b** Temperature mapping of the heating source at different input powers (~150 W, ~400 W, ~800 W, ~2000 W, from left to right). **c** Schematic of the metal powders traveling through the heating zone, the length of which is ~2 cm. **d** Simulated temperatures of 1-µm-diameter metal powders (Cr, Co, Fe, Ni, Al, Si) as a function of the travel time through the heater with a temperature of 2500 K. For all six elemental powders modeled, it takes <6 ms to reach their melting point (marked by the star symbols). **e** Simulation of the temperature of a printed NiFeCrCo MPEA half sphere (diameter = 6 mm) on a ceramic substrate during cooling.

The heater is made from carbon felt (with a size of ~10 cm × 4.5 cm × 3 mm). The center of the heater is first cut out to form a space of ~3.14 cm × 2 cm × 1 mm which is then split down the middle to form a column (diameter ~1 cm, length 2 cm). As this carbon column features higher electrical resistance, it enables the formation of a concentrated zone of Joule heating. The fabrication process for the heat-concentrated zone is shown in Supplementary Fig. 1. In a typical heating process, a constant electrical power is applied to the heater to provide Joule heating in a highly efficient manner (>90% conversion)^38^. When applying a voltage, the heat is concentrated in the column region, emitting a bright light, while the edges of the carbon felt with lower electrical resistance remain dark (Fig. 2a). We measured the temperature of the column region (the heat-concentrated area marked by the white rectangle in Fig. 2a) based on the color ratio pyrometry method using a Vision Research Phantom Miro M110 high-speed camera with video recorded at 200 frames per second^39^. The resulting temperature distribution is uniform and can be well controlled by tuning the applied power, as shown in Fig. 2b. When the applied power is ~2000 W, the temperature of the heating source (carbon strips with volume of ~0.6 cm^3^) can reach ~2500 K, and when the applied powder is ~150 W, the temperature is ~1300 K. We can even achieve temperatures as high as ~3000 K when the carbon strips are reduced to ~0.5 cm^3^ (diameter ~0.86 cm, length 2 cm) with the power tuned to ~2500 W (Supplementary Fig. 2). This wide temperature range covers the melting temperature of a large number of metallic elements, such as copper, iron, titanium, etc.

### Simulations of heating and cooling of multi-elemental metal powders

To conduct the heating process in a manner that is compatible with metal 3D printing, it is necessary to have a continuous source of the metallic feedstock. Therefore, we continuously added metal powders using a spatula suspended above the heater at a feeding rate of ~300 mg/min. Note that the flow rate can be tuned. We analyzed the transport and heating process of the metal powders in the heating zone, as illustrated in Fig. 2c. In a typical process, metal powders with a diameter of 1 µm are loaded into the vertical heating zone (set at 2500 K, length of ~2 cm) with zero initial velocity and move downwards by gravitational force. The total travel time is estimated to be ~60 ms (for simplification, only the gravitational force was considered in the calculation). Due to the high radiative heating power density, the temperature of the metal powder increases dramatically even over this short time-period. When the temperature of the metal particles reaches their melting point, the metal particles change to the liquid phase.

Based on the schematic shown in Fig. 2c, we simulated the temperature variation of six common elements, including Cr, Co, Fe, Ni, Al, and Si powders with a diameter of 1 micron as a function of the travel time and heater length when the heater temperature was 2500 K, as shown in Fig. 2d (detailed heat transfer modeling is described in the Materials and Methods and Supplementary Information). Under these conditions, the simulated results show that for all six kinds of metal powders, it takes <6 ms to reach their melting point (marked by the star symbols in Fig. 2d). As the residence time of the metal powders in the heating zone is ~60 ms, which is much longer than the time required for melting, complete melting of the powders is ensured. We also simulated the effect of the heater temperature and powder size on the temperature variation of these metal powders. When the temperature of the heater is set to 3000 K, metal powders with a diameter of 1 micron can melt into liquid in <3 ms (Supplementary Fig. 3). As further shown in Supplementary Fig. 4, at 2500 K, when the size of the metal powders is increased to 5 µm, our simulations show that it takes <12 ms for the elements to melt, which is still much less than the residence time in the heater (60 ms), further ensuring the complete melting of the metal powders during heating. As the length of the heating column can be easily tuned, it is possible to fabricate a longer heating zone to melt even larger metal powders. Complete melting of the metals is prerequisite for alloy additive manufacturing.

We also simulated the cooling process of the melted multi-elements. After being extruded from the heating zone, the melted elements are exposed to ~300 K room temperature environment. As a result, the temperature of the melted elements decreases rapidly through radiative cooling and heat convection. As an example, we simulated the cooling rate of the melted NiFeCrCo. For simplification, in the model, a melted NiFeCrCo droplet is dropped onto a ceramic substrate (5 cm × 5 cm × 1 cm) and forms a half sphere with a diameter of ~0.6 cm (Supplementary Fig. 5). The temperature of the melted NiFeCrCo as a function of time during cooling is shown in Fig. 2e. The cooling rate of the melted NiFeCrCo during the initial cooling phase (0–2 s) reaches >400 K/s. Overall, the temperature of the melted NiFeCrCo half sphere decreased from ~1760 K to ~400 K in ~10 s with an average cooling rate of 136 K/s. Such a rapid cooling rate would strongly hinder phase separation of multi-elemental products and favor homogeneous elemental distribution for uniform multi-elemental alloy formation. In addition, our heating platform has great potential to fabricate bulk metallic glass due to the high cooling rate^40^.

### Characterization of the NiFeCrCo MPEA

**Fig. 3:**
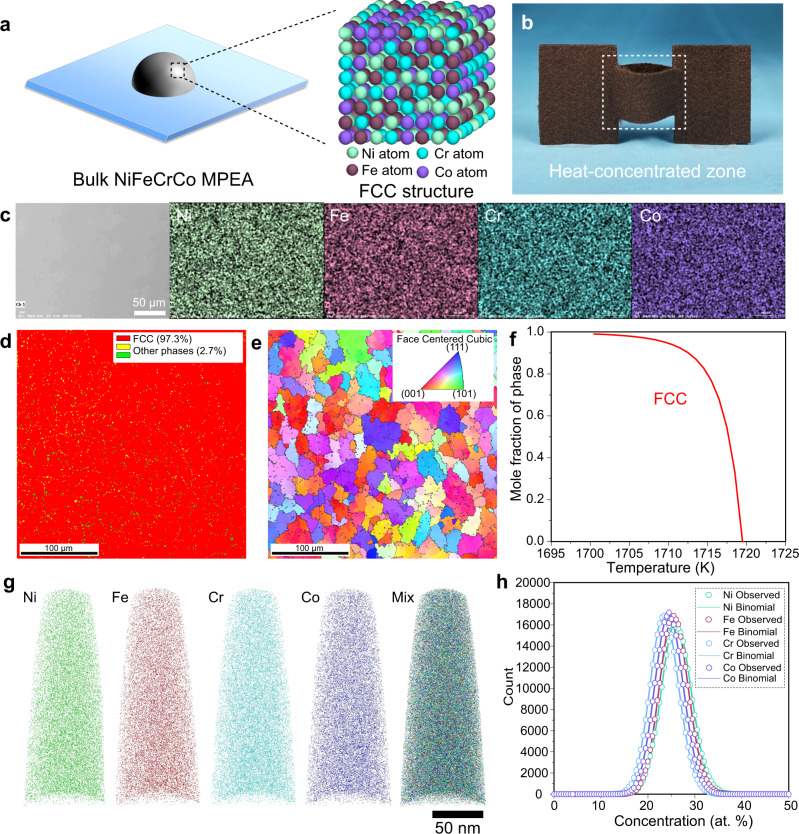
Characterization of the NiFeCrCo MPEA fabricated by the heat-concentrated, high-temperature melt printing platform. **a** Schematic of forming FCC single-phase NiFeCrCo MPEA. **b** Photograph of the heat-concentrated zone for MPEA fabrication. **c** SEM and EDS of the printed NiFeCrCo MPEA. **d** EBSD image and **e** grain size distribution of the NiFeCrCo MPEA. **f** Phase transformation path of NiFeCrCo during solidification predicted by Scheil simulation. **g** 3D APT tip reconstructions of the Ni, Fe, Cr, and Co atom positions in a typical APT tip for the fabricated NiFeCrCo MPEA. **h** Observed and statistical binomial frequency distribution analysis results of Ni, Fe, Cr, and Co for the APT tip.

As a proof-of-concept demonstration of the heating platform’s ability to rapidly mix multi-elemental metal powders for metal 3D printing, we fabricated single-phase bulk NiFeCrCo MPEA (Fig. 3a) by adding physically mixed Ni, Fe, Cr, and Co powders (diameter of ~1 micron) at equal molar ratios, which were fed into the heat-concentrated zone (Fig. 3b) set at 2500 K. The resulting droplet size of the printed alloy was ~0.6 cm, which can be controlled by tuning the size of the round opening at the bottom of the cut carbon felt heater (Supplementary Fig. 6). The microstructures of the Ni, Fe, Cr, Co powders and mixture are shown in Supplementary Fig. 7–11. To characterize the elemental distribution in the resulting NiFeCrCo MPEA, we performed scanning electron microscopy (SEM) and energy-dispersive X-ray spectroscopy (EDS) on the material’s flat polished surface. After synthesis, the mapping results show the elements thoroughly diffused and became uniformly distributed within a dense alloy phase at microscale, as shown in Fig. 3c. We also used X-ray diffraction (XRD) to characterize the crystal structure of the NiFeCrCo MPEA, which revealed an FCC dominant structure (Supplementary Fig. 12). We further characterized the phase distribution of the NiFeCrCo MPEA using electron backscatter diffraction (EBSD). As shown in Fig. 3d, the NiFeCrCo MPEA is primarily composed of the FCC phase (>97 vol.%) with other minor phases (<3 vol.%). We also measured the grain size of the FCC phase based on the EBSD results and found a uniform distribution with an average size of ~27.9 µm (Fig. 3e). With the EBSD results, different phases can be observed and determined by SEM imaging of the sample (Supplementary Fig. 13) for the identification of FCC dominant phases. We observed a relatively uniform small grain size throughout the NiFeCrCo MPEA, which is likely due to the uniform temperature distribution of the heater and rapid cooling rate, causing the MPEA grains to undergo similar temperatures and comparable growth kinetics. To further understand the impact of rapid heating and cooling on the grain size, we compared our printed NiFeCrCo MPEA sample with the same composition fabricated by arc melting, a commonly used method for the synthesis of MPEAs^41^. The arc-melted NiFeCrCo MPEA sample featured a large grain size (~188 µm, Supplementary Fig. 14), which is ~7-times larger than that of our printed sample (~27.9 µm), likely due to the comparatively slower cooling rate, which leads to grain coarsening. In order to explore the mechanism of the existence of the FCC phase, a Scheil simulation^42^ based on the Calculation of Phase Diagrams (CALPHAD) method was performed to predict the solidification path during cooling from the liquid phase. Based on the Scheil simulation, when cooling from ~1720 K, a single FCC phase is predicted to be formed and a 100% FCC phase is formed when cooling to ~1700 K (Fig. 3f), which is consistent with the experimental results.

We conducted three-dimensional atom probe tomography (3D APT) tip reconstructions (tip prepared from the NiFeCrCo MPEA) of the Ni, Fe, Cr, Co atom positions to reveal the elemental distribution at a higher resolution/at nanoscale to confirm the uniform mixing of the multi-elemental metals (Fig. 3g). Even after analyzing sixty-two million atoms, we observed no apparent elemental segregation, confirming the uniform distribution of all elements. The statistical binomial frequency distributions of all elements from the 3D APT analysis are shown in Fig. 3h. The statistical analysis shows that the NiFeCrCo MPEA tip has an overall composition of Ni_1.10_ Fe_1.09_Cr_1.00_Co_1.02_ (at. %), which is very close to the initial ratio of the metal powder precursors (Ni_1.00_Fe_1.00_Cr_1.00_Co_1.00_). The elemental distributions of Ni, Fe, Cr, and Co from the experimental results match binomial fitting curves, corresponding to a total random distribution. Overall, these results demonstrate that a NiFeCrCo MPEA with uniform elemental and grain size distribution can be fabricated using our heat-concentrated, ultrahigh-temperature platform.

### Rapid melt printing of volatile metals

**Fig. 4:**
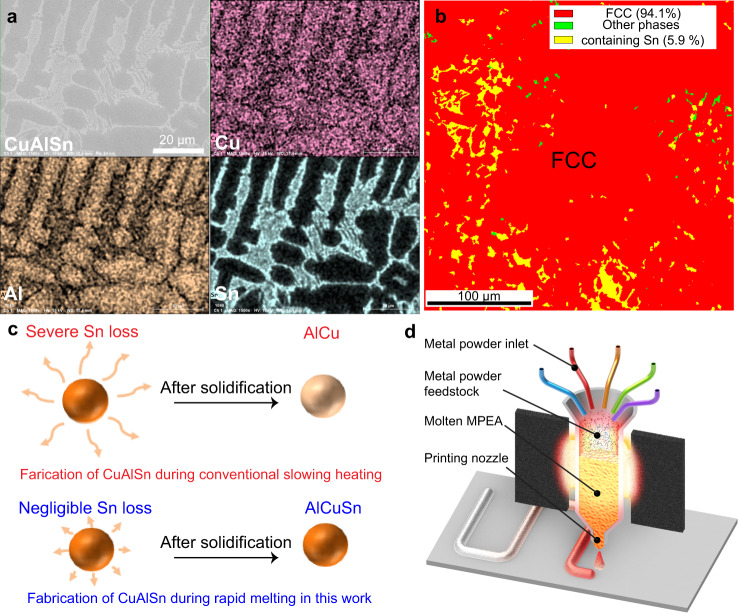
Demonstration of the minimized loss of volatile elements and schematic of the ultrahigh-temperature rapid melt printing platform as a heating source for practical 3D metal printing. **a** SEM and EDS of the CuAlSn fabricated by rapid melt printing. **b** EBSD of CuAlSn, showing a major FCC phase (~94.1 vol.%) and a small fraction of minor phases containing Sn (~5.9 vol.%). **c** Schematics of the fabrication of the CuAlSn alloy by slow heating with severe Sn loss compared to the negligible Sn loss during rapid melt printing. **d** Schematic of practical MPEA 3D printing using our ultrahigh-temperature rapid melt printing platform as a heating source.

Besides the rapid mixing of multi-principal elements, the short melting time can also reduce the loss of volatile metals. For example, setting the heater to ~2000 K, we printed a CuAlSn alloy (mass ratio = 90:4:6), which features a large difference in the melting points of the constituent elements. Specifically, Sn and Al are volatile with low melting temperatures (~500 K and 933 K, respectively), which are ~850 K and ~420 K lower than that of Cu (~1357 K)^43–45^. As a result, traditional fabrication of CuAlSn may lead to severe evaporative loss of Sn while attempting to achieve complete melting of Cu^46^. Using our heat-concentrated platform to synthesize the CuAlSn alloy, we observed the co-existence of two major phases (CuAl-rich phase and Sn-rich phase), as shown by SEM and EDS analysis in Fig. 4a. Within each phase, all the elements are distributed uniformly. In addition, the elemental analysis of the final product shows the CuAlSn has an overall composition of Cu_90.3_Al_3.8_Sn_5.9_ (wt.%), which is very close to the initial ratio of the metal powder precursors (Cu_90_Al_4_Sn_6_), as shown in Supplementary Table 1. Along with the EBSD analysis (Fig. 4b, Supplementary Fig. 15), we conclude the Al and Cu alloyed into a major FCC phase (~94.1 vol.%), with a small fraction of other minor phases containing Sn (~5.9 vol.%). This is consistent with the XRD result that demonstrates FCC-phase dominant patterns (Supplementary Fig. 16).

For comparison, we also fabricated the CuAlSn alloy by traditional arc melting and found the sample suffered severe Sn loss (~30 wt.%), with a composition of Cu_92_Al_4_Sn_4_ (Supplementary Table 1) and a significantly reduced amount of Sn-containing phase due to the volatile nature of Sn as well as possible loss due to fly-off of the powders when the arc strikes over the raw material (Supplementary Fig. 17). In contrast, our rapid melt printing method was able to successfully fabricate bulk CuAlSn as we have negligible Sn loss during the rapid melting process (Fig. 4c). Due to the combination of the sufficiently high temperature and uniform temperature distribution of the heating zone, along with the minimized loss of volatile elements, our rapid melt printing platform demonstrates great potential as a heating source for practical 3D metal printing of a wide range of MPEAs. As schematically shown in Fig. 4d, multi-elemental metal powders could be loaded automatically into an ultrahigh-temperature stable printhead (e.g., tungsten) wrapped with the carbon felt heater to serve as the concentrated heating source for continuous MPEA printing. Based on the electrified Joule heating principle, the heater features high temporal resolution (1 ms) and wide temperature (up to 3000 K) and cooling rate (up to 10^5^ K/s) range^47^. Therefore, we expect this technique to be complementary to state-of-the-art melting techniques such as electron beam, laser, and wire arc-melting, commonly used for additive manufacturing.

## Discussion

To solve the tradeoff of achieving both high heating temperature and a sufficient heating zone with uniform temperature distribution for the fabrication of MPEAs with high homogeneity towards 3D printing, we designed a high-temperature, rapid melt printing platform which demonstrates great potential for MPEA additive manufacturing. Specifically, we designed a concentrated-heating zone using a piece of carbon felt that had been cut to form a column that featured high electrical resistance for high-temperature Joule heating. In a typical printing process, micron-sized metal powders are loaded continuously into the heat-concentrated zone, which can be heated to as high as 3000 K. The sufficient high temperature and relatively large heating zone with uniform temperature distribution simultaneously enables rapid melting, mixing, and alloying of multi-elemental metal powders on the order of milliseconds to produce MPEAs with high homogeneity. As proof-of-concept, we successfully fabricated single-phase dominated NiFeCrCo MPEA. Besides the rapid melting rate, the fabricated MPEA can also be rapidly cooled at a rate of ~10^2^ K/s, which is beneficial for a homogenous multi-elemental chemical composition and microstructure with uniform grain size. Our rapid melt heating platform features several advantages, including (1) a concentrated high-temperature zone (up to 3000 K), enabling a broad range of metals to be used; (2) a relatively large heating zone with rapid and uniform melting and mixing of multiple metals into high density bulk MPEA materials with uniform grain size; (3) minimized loss of volatile elements due to the rapid heating process; and (4) suitability for high-throughput screening of different MPEA compositions for a wide range of applications. All the above advantages suggest the strong potential of our rapid melt printing platform as a heating source for MPEA 3D printing.

## Methods

### High-temperature heater

The heat-concentrated zone is made from carbon felt (size of ~10 cm × 4.5 cm × 3 mm). The center of the heater is first cut to form a smaller area of ~3.14 cm × 2 cm × 1 mm, which is then split down the middle to form an open column space (diameter ~1 cm, length 2 cm) that extends down through the middle of the felt. By employing two copper clips, the carbon heater is connected to a high-power DC source (Volteq HY6020EX) with tunable current (0–50 A) and voltage (0–50 V) and heated in an argon atmosphere.

### Multi-elemental metal powders for MPEA fabrication

The precursor metal powders were purchased from Atlantic Equipment Engineers, including Fe (99.9 wt.%, ~1 μm), Ni (99.9 wt.%, ~1 μm), Cr (99.9 wt.%, ~1 μm), Co (99.9 wt.%, ~0.5 μm), Al (99.9 wt.%, 1–5 μm), Cu (99.9 wt.%, 1–5 μm), and Sn (99.9 wt.%, 1–5 μm). Stoichiometric amounts of the elemental powders were uniformly mixed by milling for 30 min. The mixed powders were then loaded into the column of the high-temperature heater by a spatula for fabrication of the bulk alloy.

### Arc melting fabrication of the NiFeCrCo MPEA and CuAlSn alloy

To fabricate the NiFeCrCo MPEA by arc melting, a button weighing 10 g with a diameter of ~1 cm was cast using an arc melter (ABJ-338, Materials Research Furnaces Inc., USA) with 2.5 g of each constituent element. For the fabrication of the CuAlSn alloy, 9 g of Cu, 0.4 g of Al, and 0.6 g of Sn were arc melted into a CuAlSn button using the same equipment.

### Material characterization

The printed alloy samples were flat polished by metallographic polishing steps with a 0.04 µm silica suspension as the final step. The microstructure and morphology of the prepared samples with flat polished surfaces were measured using a Hitachi SU-70 FEG-SEM at 10 kV. EBSD was conducted on an FEI Scios Dual-Beam system with an accelerating voltage of 20 kV. The scan areas were 300 × 300 µm on the surface of the polished samples and the step sizes were ~0.5–0.7 µm. The 3D APT experiments were performed utilizing a local-electrode atom-probe tomograph (CAMECA LEAP5000XS), with a pulse laser energy of 20 pJ, a pulse frequency of 250 kHz and a specimen temperature of 50 K. The APT nano-tips were prepared using a two-step electropolishing process on rods center-cut from the printed NiFeCrCo MPEA sample by wire electrical discharge machining (EDM). Initial electropolishing was performed with a solution of 10 vol.% perchloric acid in acetic acid at 10–15 V dc at room temperature. Final sharpening was done in a second, weaker solution of 2% perchloric acid in 2-butoxyethanol at 10–25 V dc. Analyses of the 3D APT data were carried out using the IVAS 3.8 software package (Cameca, Madison, WI).

### Heat transfer modeling

When a µm-scale metal particle travels in the vertical heating zone, the temperature of the particle increases rapidly because of radiative heating. When the temperature of the metal particle reaches its melting point, the metal particle changes to the liquid phase and evaporation occurs. Here, we employed a heat transfer model to investigate the heating process of metal particles in the heating zone. For simplicity, we made the following assumptions: (1) the metal particles are spherical, and the size of the metal particles is uniform; (2) the particles are uniformly distributed in the heating zone; (3) the convective heat transfer coefficient is ${h}_{c}\, \approx \, 0{{{{{\rm{W}}}}}}/\left({{{{{{\rm{m}}}}}}}^{2}\, \bullet \,{{{{{\rm{K}}}}}}\right)$ due to the low velocity of the metal particles (<0.4 m/s); (4) the metal particles are only heated by the thermal radiation of the heater, and the radiative heating from the surrounding metal particles is ignored; (5) the size of the particles remains constant after they change to the liquid phase; (6) the evaporation of the materials can be ignored before the particle reaches its boiling point; (7) the size effect of the thermophysical properties of the µm-scale metal particles can be ignored; (8) the optical properties (e.g., surface emissivity and complex refractive index) of the metal particles and heaters are independent of the temperature. Therefore, the heating process of a spherical particle, which absorbs radiative energy, can be described by^48^,

1 $${\rho }_{p}{C}_{p}\left({T}_{p}\right)\frac{\partial {T}_{p}}{\partial t}={k}_{p}\left({T}_{p}\right)\frac{1}{{r}^{2}}\frac{\partial }{\partial r}\left({r}^{2}\frac{\partial {T}_{p}}{\partial r}\right)+{q}_{{rad}}-\triangle H\left({T}_{p}={T}_{{{{{{\rm{melt}}}}}}}\right)$$

with the initial condition,

$${T}_{p}\left(t=0\right)={T}_{0}$$

where ${\rho }_{p}$, ${C}_{p}\left({T}_{p}\right)$, and ${T}_{p}$ are the density, heat capacity, and temperature of the metal particles, respectively, ${k}_{p}$ denotes the thermal conductivity of the metal particles, ${T}_{0}=300{{{{{\rm{K}}}}}}$ is the room temperature, $\triangle H$ is the enthalpy of melting, and ${q}_{{rad}}$ is the radiative heat received from the heater, which can be described by,

2 $${q}_{{rad}}={\int }_{0}^{{{\infty }}}{\varepsilon }_{p}\left(\lambda \right)S\left(\lambda \right){\varepsilon }_{h}\left(\lambda \right){E}_{h}\left({T}_{h},\, \lambda \right)d\lambda -{\int }_{0}^{{{\infty }}}{\varepsilon }_{p}\left(\lambda \right){E}_{p}\left({T}_{p},\, \lambda \right)d\lambda$$

where $\lambda$ is the wavelength, ${\varepsilon }_{p}\left(\lambda \right)$ and ${\varepsilon }_{h}\left(\lambda \right)$ are the surface emissivities of the metal particles and heater, $S\left(\lambda \right)$ is the transmission factor, which accounts for the attenuation of the thermal radiation emitted by the heater by the dispersed metal particles (Supplementary Figs. 18–20, Supplementary Table 2, Supplementary Note 1, and Supplementary Eqs. (1–5)), ${E}_{h}\left({T}_{h},\, \lambda \right)$ and ${E}_{p}({T}_{p},\, \lambda )$ are the spectral black body radiation of the heater and metal particles at temperature ${T}_{h}$ and ${T}_{p}$, respectively, which can be evaluated as^48^,

3 $${E}_{\lambda }=\frac{2h{c}^{2}}{{\lambda }^{5}}\frac{1}{{e}^{\frac{{hc}}{{k}_{b}T\lambda }}-1}$$

where $h$ is Planck’s constant, $c$is the speed of light, and ${k}_{b}$ is the Boltzmann constant. The temperature-dependent thermal conductivity, heat capacity, and emissivity are shown in Supplementary Figs. 21–23.

## Supplementary information

Supplementary Information

## Data Availability

The data that support the findings of this study are available within this article and its Supplementary Information. Source data are provided with this paper.

## Source data

Source Data
