# Publisher Correction: Ultrahigh-temperature melt printing of multi-principal element alloys

**DOI:** 10.1038/s41467-022-35053-3

**Published:** 2022-11-30

**Authors:** Xizheng Wang, Yunhao Zhao, Gang Chen, Xinpeng Zhao, Chuan Liu, Soumya Sridar, Luis Fernando Ladinos Pizano, Shuke Li, Alexandra H. Brozena, Miao Guo, Hanlei Zhang, Yuankang Wang, Wei Xiong, Liangbing Hu

**Affiliations:** 1 Department of Materials Science and Engineering, University of Maryland, College Park, Maryland 20742 USA; 2 Center for Materials Innovation, University of Maryland, College Park, Maryland, 20742, USA; 3 Department of Mechanical Engineering and Materials Science, University of Pittsburgh, Pittsburgh, PA 15261 USA; 4 Center for Hierarchical Materials Design, Northwestern University, Evanston, IL 60208 USA

**Keywords:** Design, synthesis and processing, Metals and alloys

Correction to: *Nature Communications* 10.1038/s41467-022-34471-7, published online 07 November 2022

The original version of this Article contained an error in the affiliation of the 13^th^ author, Wei Xiong from ‘Department of Mechanical Engineering and Materials Science, University of Pittsburgh, Pittsburgh, PA 15261, USA’, which was incorrectly given as ‘Center for Materials Innovation, University of Maryland, College Park, Maryland, 20742, USA’. The error has been corrected in both the PDF and HTML versions of the Article.

