# Supplementary material for: Ultrahigh-temperature melt printing of multi-principal element alloys
Source: Nat Commun. 2022 Nov 7;13:6724. doi: 10.1038/s41467-022-34471-7 (PMC9640643; doi:10.1038/s41467-022-34471-7)
Supplement: Supplementary file 1 — Supplementary Information [file 41467_2022_34471_MOESM1_ESM.pdf]

Supplementary Information

Ultrahigh-Temperature Melt Printing of Multi-Principal Element Alloys

Xizheng Wang,[1,*] Yunhao Zhao,[2,*] Gang Chen,[1,*] Xinpeng Zhao,[1,*] Chuan Liu,[3] Soumya Sridar,[2] Luis Fernando Ladinos Pizano,[2] Shuke Li,[1] Alexandra H. Brozena,[1] Miao Guo,[1] Hanlei Zhang,[2] Yuankang Wang,[2] Wei Xiong,[2,†] Liangbing Hu[1,†]

1. Department of Materials Science and Engineering, University of Maryland, College Park, Maryland, 20742, USA
2. Department of Mechanical Engineering and Materials Science, University of Pittsburgh, Pittsburgh, Pennsylvania, 15260, USA
3. Center for Hierarchical Materials Design, Northwestern University, Evanston, IL 60208, USA

[†] Corresponding author. Email address: binghu@umd.edu; weixiong@pitt.edu

[*] These authors contributed equally to this work.

Table of Contents

Supplementary Fig. 1-23

Supplementary Table 1-2

Supplementary Note 1

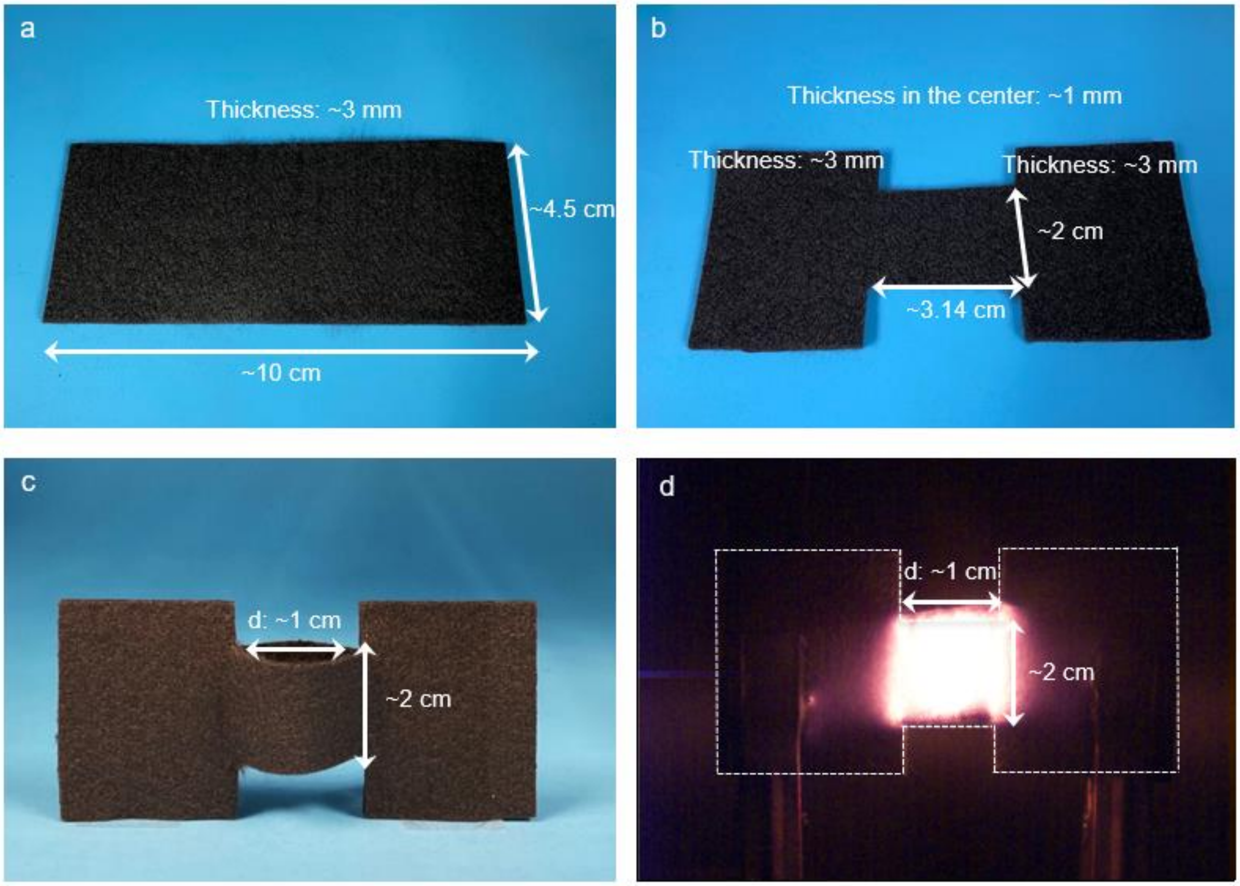

Supplementary Fig. 1. Fabrication process of the heat-concentrated zone from carbon felt. a Photograph of the carbon felt starting material, with a size of ~10 cm × 4.5 cm × 3 mm. **b** Photograph of the center of the heater cut out to form an area of about 3.14 cm × 2 cm × 1 mm. **c** Photograph of the center of the heater split down the middle to form an open column space (diameter ~1 cm, length 2 cm). **d** Photograph of the heater under Joule heating. Due to the higher electrical resistance of the carbon column, a Joule heating-concentrated zone is consequentially formed.

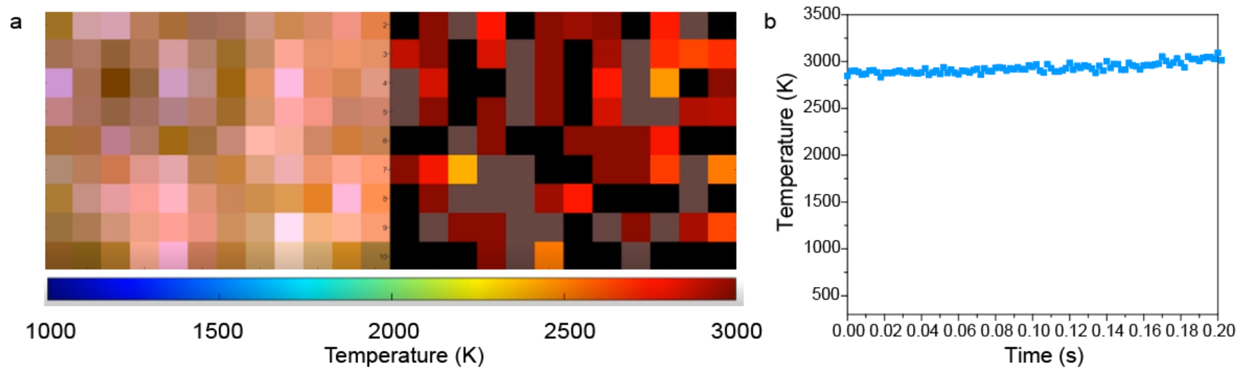

Supplementary Fig. 2. Temperature characterization of the heat-concentrated zone, which can reach ~3000 K. a Temperature mapping of the heater at 3000 K. **b** Temperature profile of the heater that can reach as high as ~3000 K.

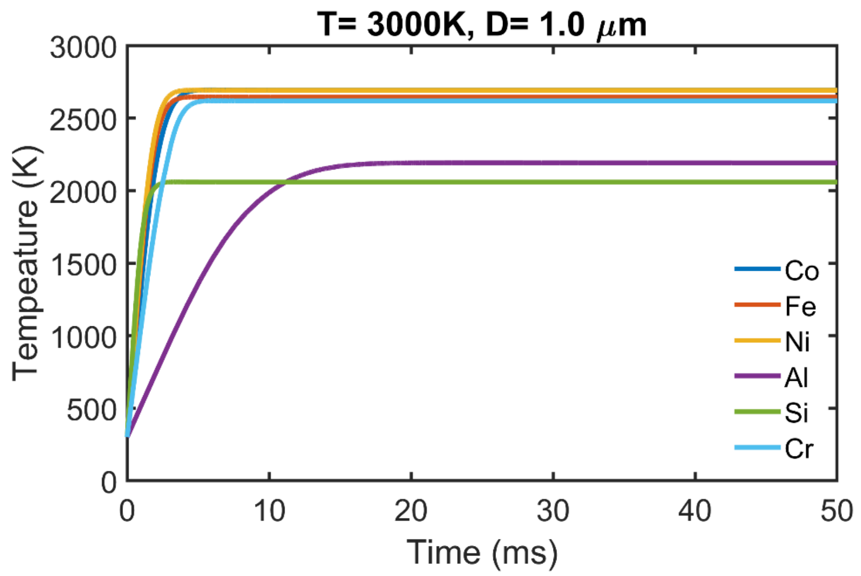

Supplementary Fig. 3. Simulated heating temperatures of different elemental powders (Fe, Co, Ni, Al, Si, Cr) of 1 micron in diameter in the center of the heating region vs. the traveling time in the heater at 3000 K. When the temperature of the heater is set to 3000 K, the metal powders with a diameter of 1 micron can melt into liquid in less than 3 ms.

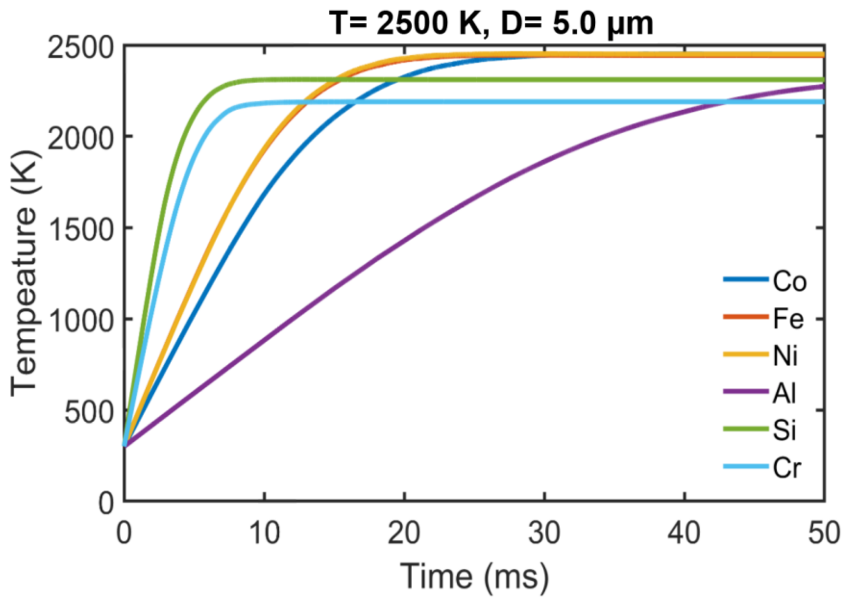

Supplementary Fig. 4. Simulated heating temperature of different elemental powders (Fe, Co, Ni, Al, Si, Cr) of 5 microns in diameter in the center of the heating region vs. the traveling time in the heater at 2500 K. At 2500 K, when the size of the metal powders is increased to 5 μm, it takes no more than 12 ms for the six different elements to melt, which is much less than the residence time in the heater (60 ms), ensuring complete melting during heating.

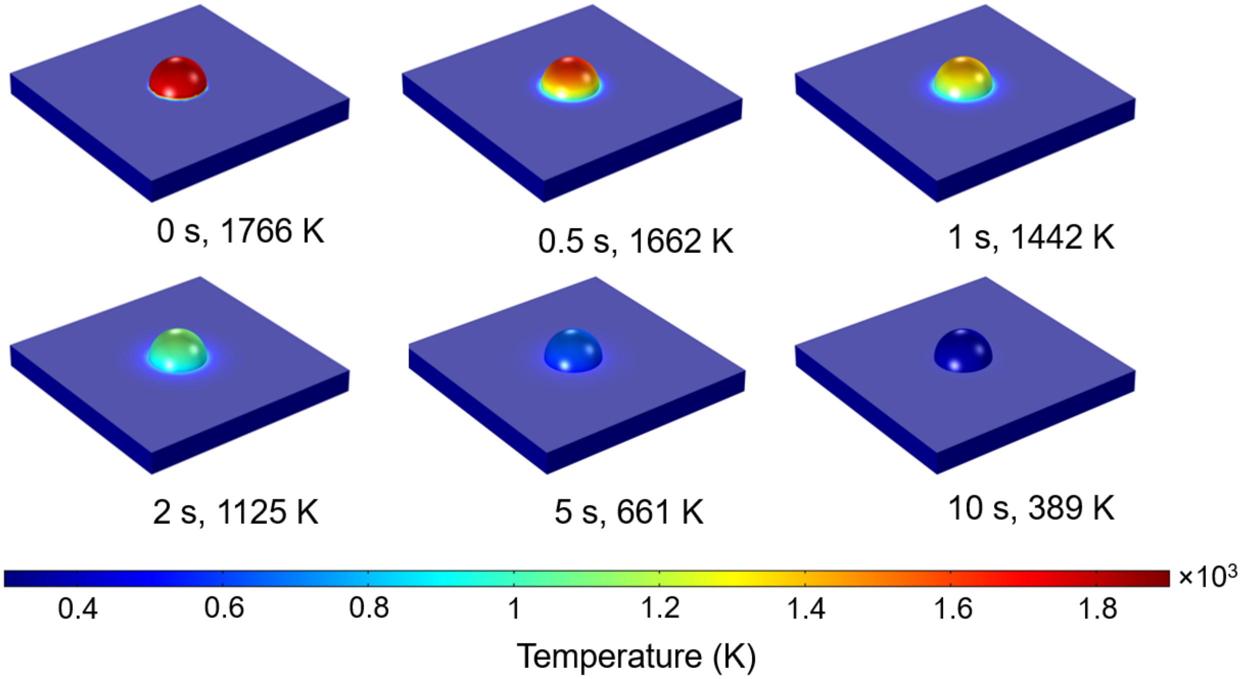

Supplementary Fig. 5. Simulated cooling rate of the melted NiFeCrCo. For simplification, in the model, a melted NiFeCrCo droplet was added onto a ceramic substrate (5 cm×5cm×1cm) and formed a half sphere with a diameter of ~0.6 cm. The simulation was performed using COMSOL Multiphysics. The cooling rate of the melted NiFeCrCo during the initial cooling phase (0–2 s) reaches > 400 K/s. Overall, the temperature of the melted NiFeCrCo decreased from ~1760 K to ~400 K in ~ 10 s with an average cooling rate of 136 K/s.

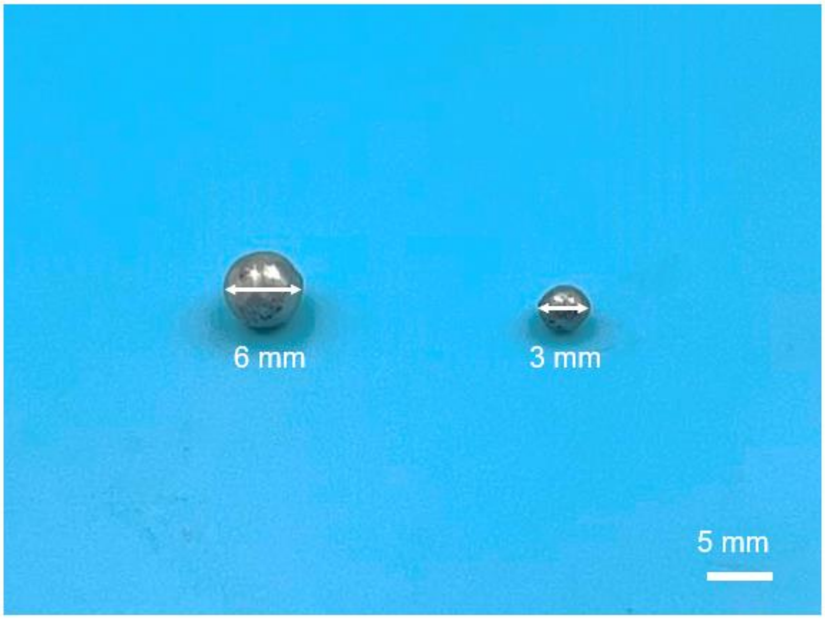

Supplementary Fig. 6. Digital images of the printed NiFeCrCo MPEA droplets with diameter of 6 mm and 3 mm. We obtained droplets of ~6 mm in diameter using a carbon felt heater with an outlet hole cut to a similar size. By further decreasing the size of the column opening in the carbon felt heater to ~3 mm, we obtained smaller droplets of ~3 mm in size.

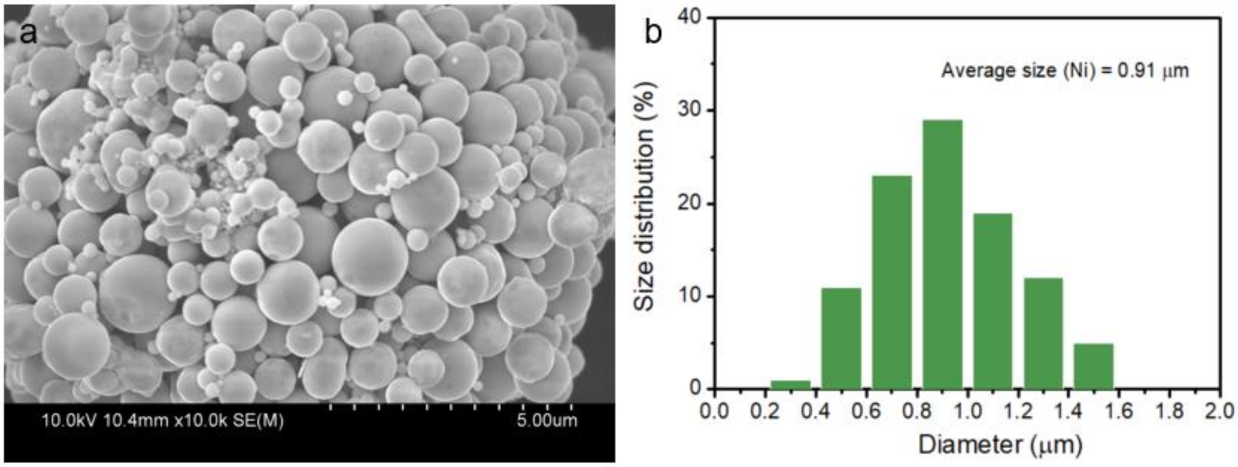

Supplementary Fig. 7. SEM of the Ni powder and size distribution. a SEM of the Ni powder. **b** The Ni powder size distribution. The average size of the Ni powder was ~1 μm.

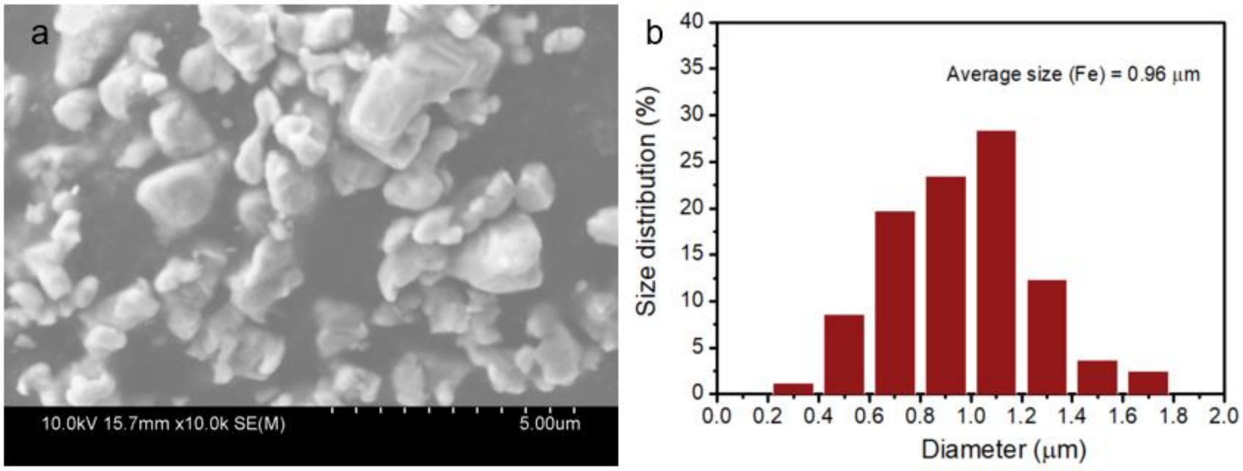

Supplementary Fig. 8. SEM of the Fe powder and size distribution. a SEM of the Fe powder. **b** The Fe powder size distribution. The average size of the Fe powder was ~1 µm.

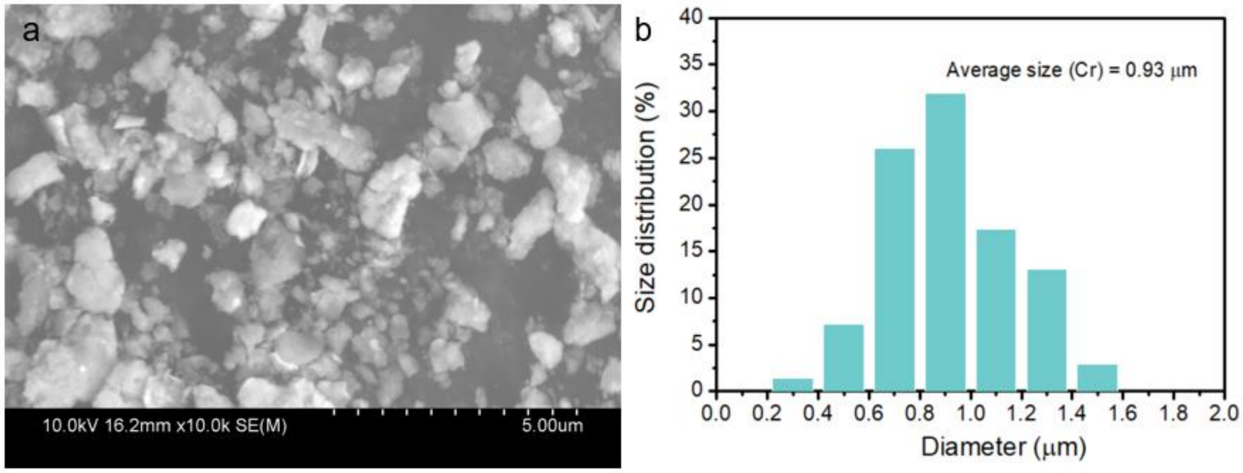

Supplementary Fig. 9. SEM of the Cr powder and size distribution. a SEM of the Cr powder. **b** The Cr powder size distribution. The average size of the Cr powder was ~1 µm.

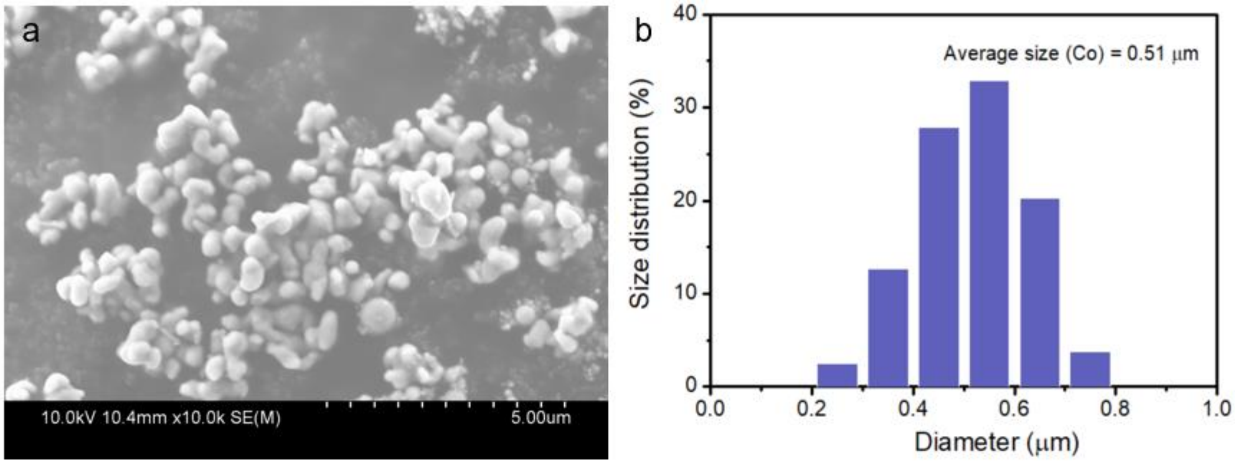

Supplementary Fig. 10. SEM of the Co powder and size distribution. a SEM of the Co powder. **b** The Co powder size distribution. The average size of the Co powder was ~0.5 µm.

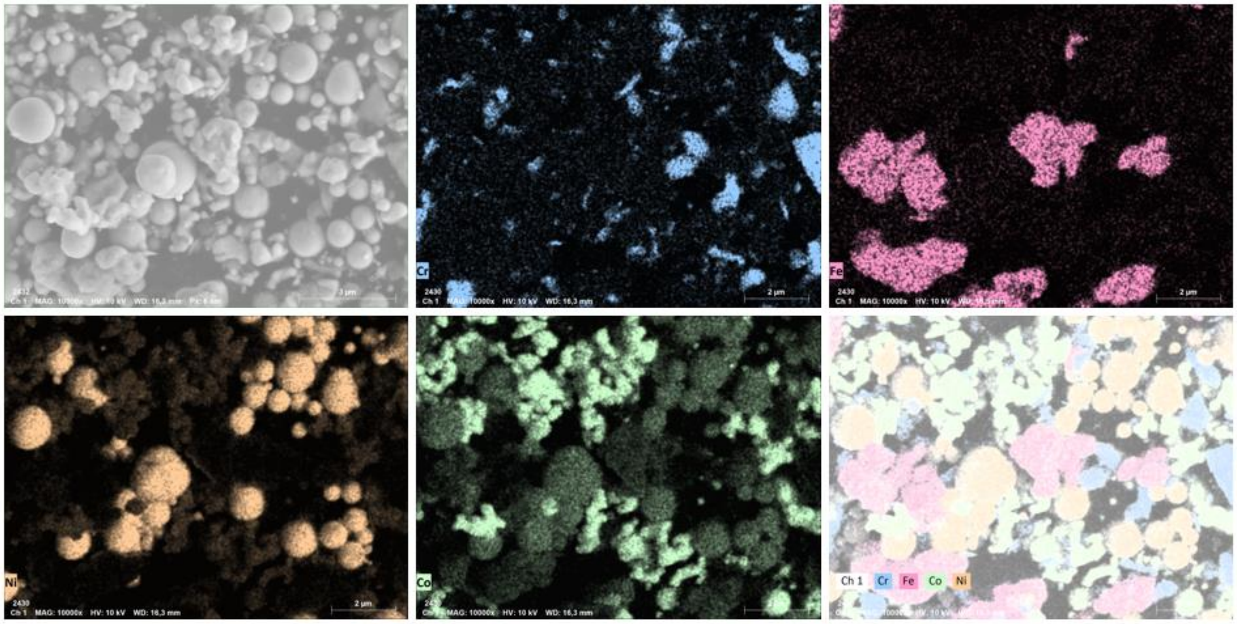

Supplementary Fig. 11. SEM and EDS of the mixed NiFeCrCo powder. The elements are uniformly mixed in the powder.

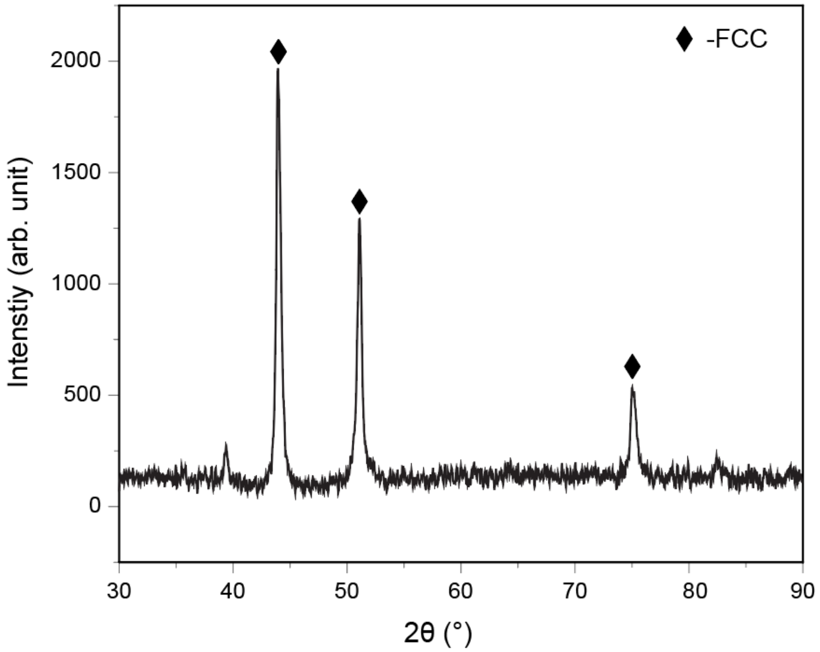

Supplementary Fig. 12. XRD of the printed NiFeCrCo MPEA, fabricated by the high-temperature melt printing platform, showing an FCC dominant structure.

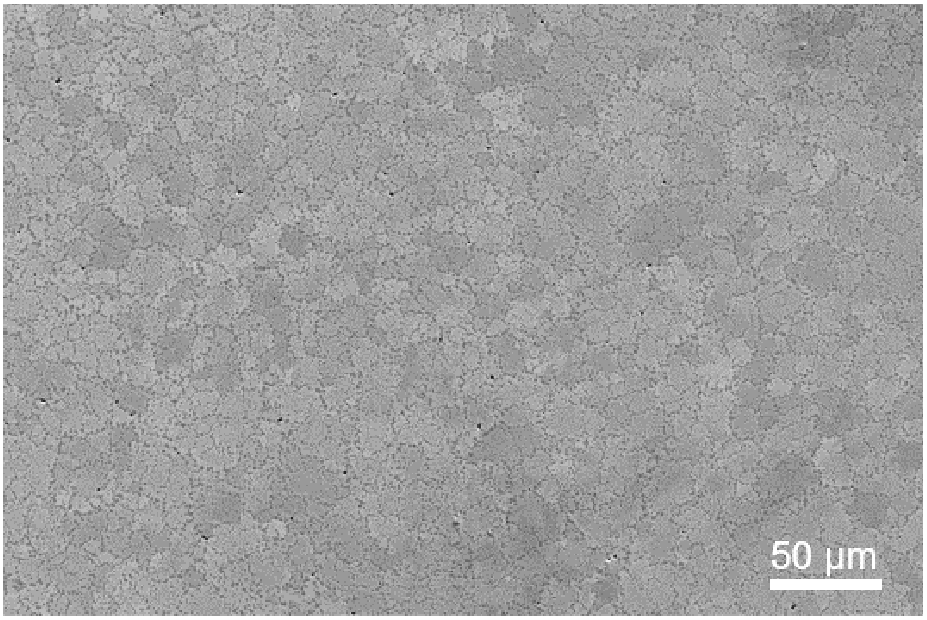

Supplementary Fig. 13. SEM of the NiFeCrCo MPEA by rapid melt printing at high magnification (compared to Fig. 3c). The large proportion of light-grey and mid-grey areas appear to match with the FCC phase labeled in EBSD (Fig. 3e).

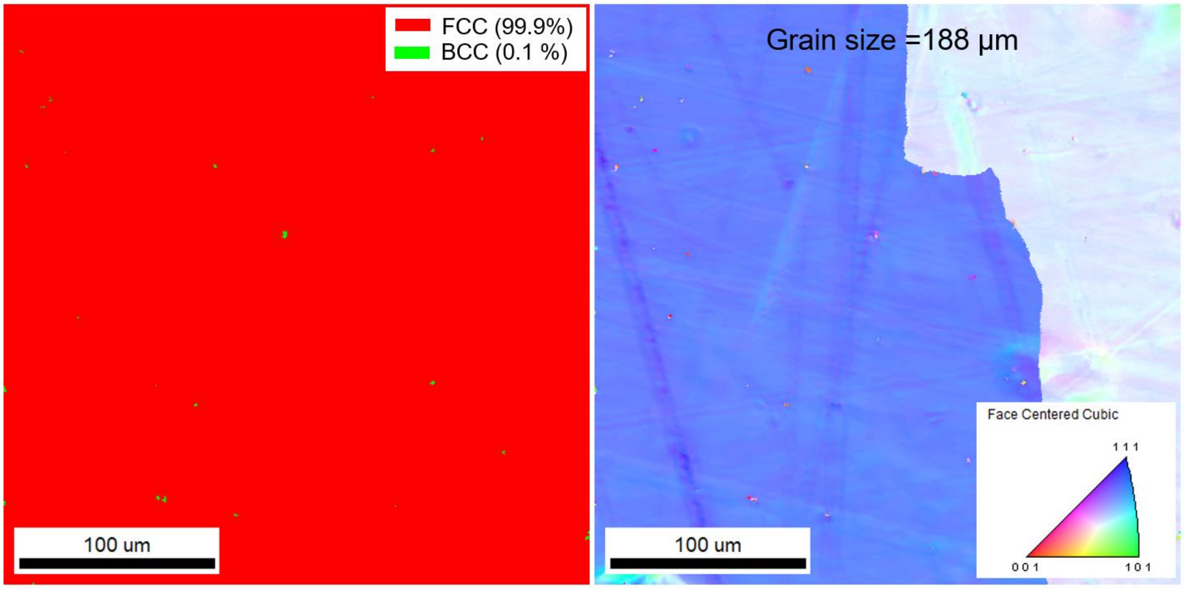

Supplementary Fig. 14. EBSD image and grain size distribution of the arc-melted NiFeCrCo MPEA, which features a significantly large grain size (~188 µm).

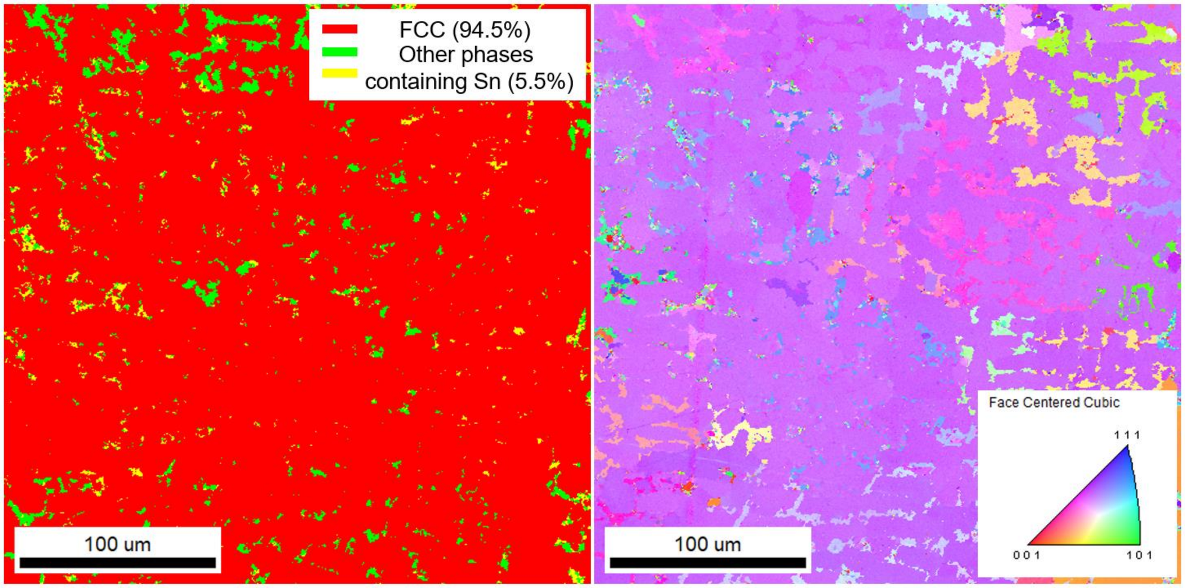

Supplementary Fig. 15. EBSD and grain size distribution of the printed CuAlSn alloy fabricated by the high-temperature melt printing platform. Al and Cu alloyed into a major FCC phase (~94.5 vol.%), with a small fraction of other minor phases containing Sn (~5.5 vol.%).

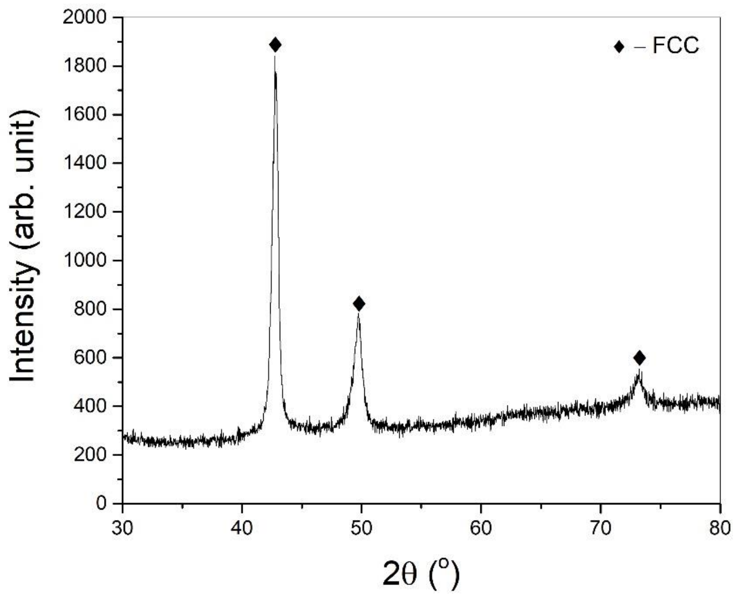

Supplementary Fig. 16. XRD of the printed CuAlSn alloy fabricated by the high-temperature melt printing platform, showing an FCC dominant structure.

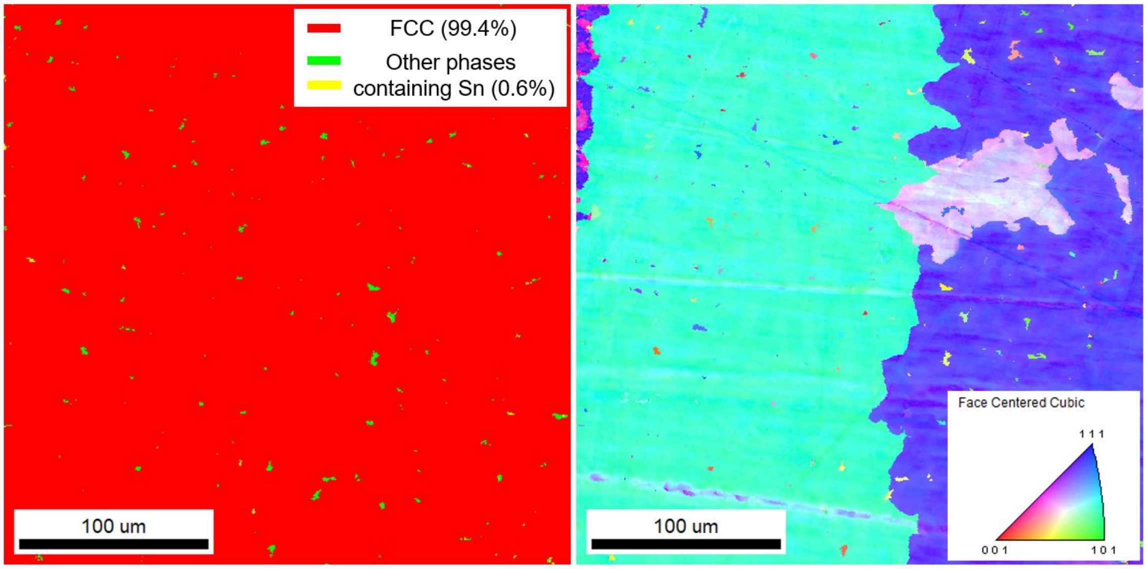

Supplementary Fig. 17. EBSD image and grain size distribution of the arc-melted CuAlSn, demonstrating severe Sn loss. The proportion of Sn containing phases (~0.6%) is significantly lower than that of the melt-printed sample shown in Fig. S15.

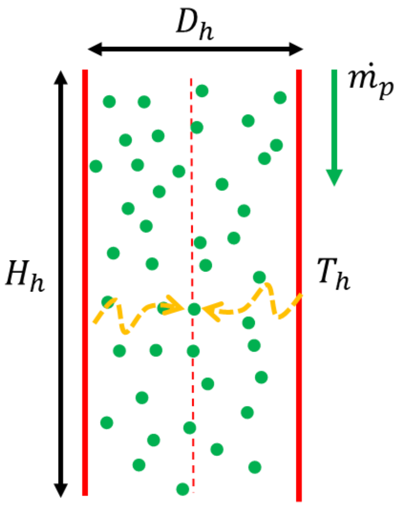

Supplementary Fig. 18. Schematic of the metal particles traveling in the heating zone, where the diameter and length of the heater is D_h and H_h, respectively, the temperature of the wall is T_h, and the flow rate of the metal particles is $\dot{m}_p$.

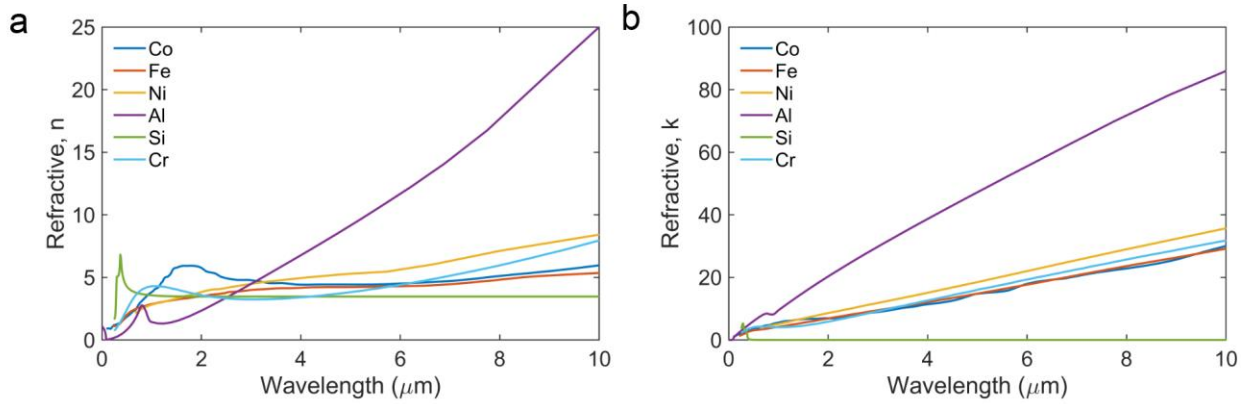

Supplementary Fig. 19. Spectral refractive indices of different metal particles (Fe, Co, Ni, Al, Si, Cr); (a) real part, (b) imaginary part[9–13]. The refractive indices are adopted to simulate the transmission of the metal particles traveling in the heating zone.

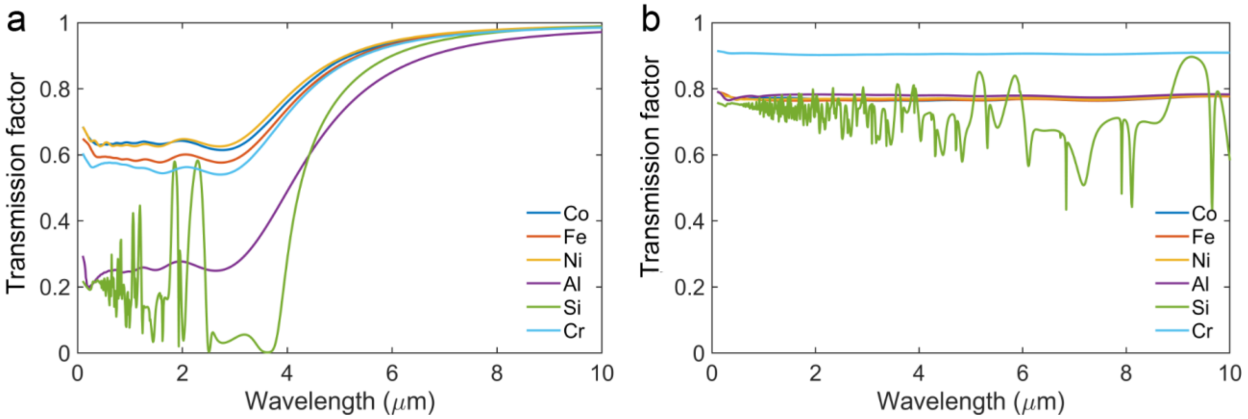

Supplementary Fig. 20. Transmission factor of the metal particles traveling along the central line of the heating zone for particle diameters (D_p) of (a) 1 μm and (b) 5 μm. The feeding rate of the metallic powders was 300 mg/min. As metal particle size decreases, the transmission factor decreases since the diameter of small size particles (i.e., 1 μm) is more comparable to the wavelength of high temperature radiation.

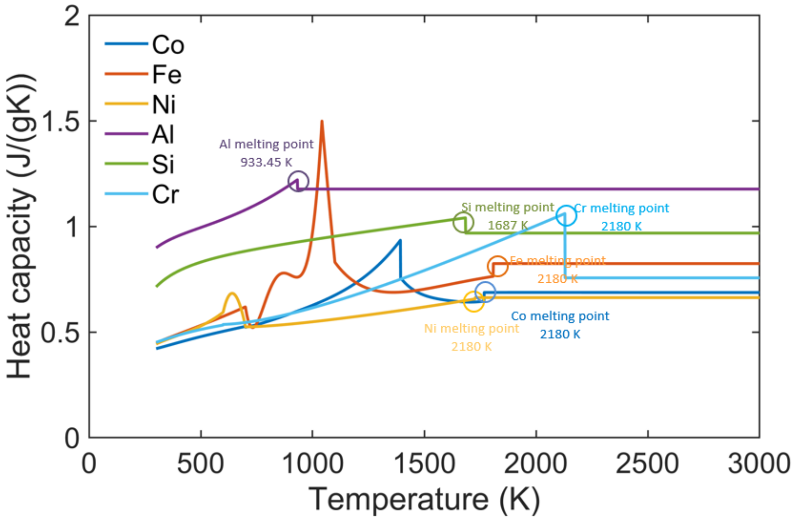

Supplementary Fig. 21. Temperature-dependent heat capacity of different elemental powders (Fe, Co, Ni, Al, Si, Cr)[1]. The heat capacities were used as the input for simulating the heating process of the metal particles traveling in the heating zone.

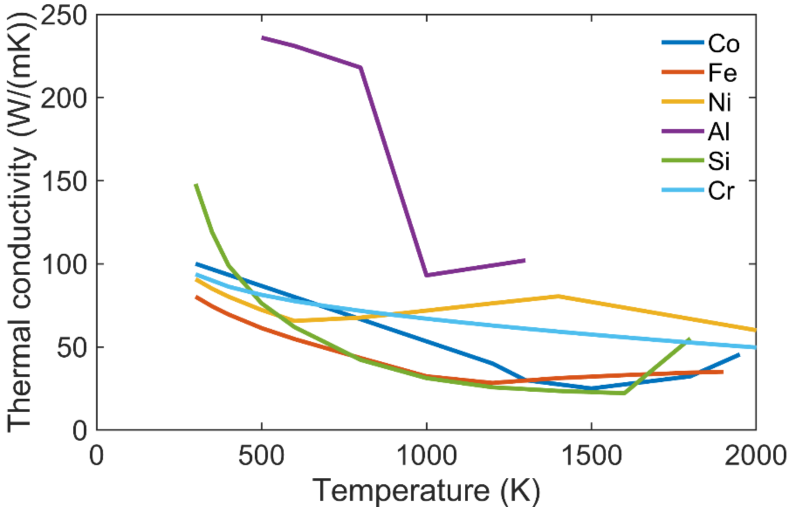

Supplementary Fig. 22. Temperature-dependent thermal conductivity of different elemental powders (Fe, Co, Ni, Al, Si, Cr)[2–5]**.** The thermal conductivities were used as the input for simulating the heating process of the metal particles traveling in the heating zone.

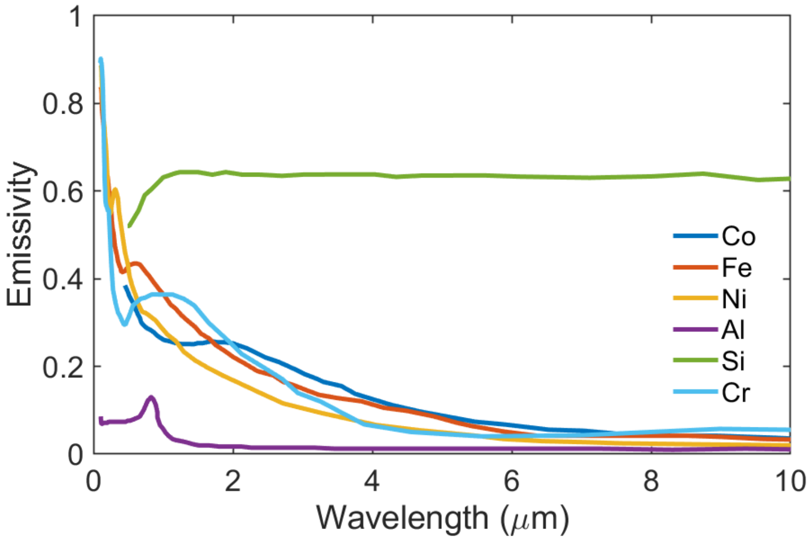

Supplementary Fig. 23. Spectral emissivity of different elemental powders (Fe, Co, Ni, Al, Si, Cr)[6,7]**.** The spectral emissivities were used as the input for simulating the heating process of the metal particles traveling in the heating zone.

Supplementary Table 1. The composition of CuAlSn before and after rapid melt printing and arc melting. The CuAlSn alloy produced by rapid melt printing has an overall composition of $Cu_{90.3}Al_{3.8}Sn_{5.9}$ (wt.%), which is very close to the initial ratio of the metal powder precursors ($Cu_{90}Al_{4}Sn_{6}$). The arc-melted CuAlSn sample suffers severe Sn loss (~30 wt.%), with a composition of $Cu_{92}Al_{4}Sn_{4}$.

	Composition (in wt.%)		
	Cu	Al	Sn
Initial ratio (before melting)	90	4	6
Rapid melt printing	90.3	3.8	5.9
Arc melting	92.0	3.8	4.2

Supplementary Table 2. Materials studied and their corresponding physical and thermodynamic properties.

Property	Density (solid)	Melting Temperature	Density (liquid)	Boiling Temperature	Enthalpy (S to L)
Unit	kg/m^3	K	kg/m^3	K	J/g
Cobalt	8900	1768	8860	3143	305
Iron	7860	1608	6980	3134	222
Nickel	8900	1728	7810	3003	298
Aluminum	2710	933	2375	2743	391
Silicon	2330	1687	2570	3538	1726
Chromium	7190	2180	6300	2945	501
NiFeCrCo	8183				

Supplementary Note 1

As shown in Supplementary Fig. 18, the radiative heating process of the metal particles can be simplified by assuming a dilute particle flow in a cylindrical heating zone with a wall temperature of T_h. The metal particles with an ignorable initial velocity are first introduced at the top of the heating zone; then, they flow through the heating region driven by gravity (flow time is $t_p = \sqrt{2H_h/g}$, where g is the gravitational constant). Since the flow rate is very low (< 0.4 m/s), we only considered radiative heating in our work. Due to the diameter of the µm-scale metal particles being close to the peak wavelength of the black body emission of the high-temperature heater (e.g., 2500 K), the thermal radiation emitted by the heater will be attenuated by the dispersed particles, resulting in a lower heating rate. With the assumption of the random distribution and low volume fraction (< 0.01%) of the metal particles in the heating zone, each metal particle can be treated as an individual scattering center. Therefore, the scattering (Q_{sca}), extinction (Q_{ext}), and absorption (Q_{abs}) efficiency factors can be evaluated by Lorenz-Mie theory[8],

$$Q_{sca,\lambda} = \frac{2}{x^2}\sum_{l=1}^{\infty}(2l+1)\left(|a_l|^2 + |b_l|^2\right) \tag{1}$$

$$Q_{ext,\lambda} = \frac{2}{x^2}\sum_{l=1}^{\infty}(2l+1)\,Re(a_l + b_l) \tag{2}$$

$$Q_{abs,\lambda} = Q_{ext,\lambda} - Q_{sca,\lambda} \tag{3}$$

where $x = \pi D_p/\lambda$, l is an integer, a_l and b_l are Mie coefficients as a function of x and complex refractive index (i.e., $m = n + ik$, see Supplementary Fig. 19).

The spectral extinction coefficient of the dispersed metal particles in the heating zone can be estimated by,

$$\beta(\lambda) = \frac{3}{2}\frac{V_f Q_{ext,\lambda}}{D_p} \tag{4}$$

where $V_f = 4\dot{m}_p t_p/\rho_p \pi D_h^2 H_h$ is the volume fraction of the metal particles in the heating zone and ρ_p is the density of the metal particles (Supplementary Table 2). To estimate the heating time of the metal particles, we modeled the heating process of the metal particles that travel along the central line of the cylindrical heating zone (dashed line of Supplementary Fig. 18) as these particles should undergo the lowest transmission factor with the longest melting time. The spectral transmission factor (Supplementary Fig. 20), which evaluated the attenuation of the thermal radiation emitted by the heater, can be determined based on Beer's law[14],

$$S(\lambda) = e^{-\frac{\beta(\lambda) D_h}{2}} \tag{5}$$

We see that the transmission factor of Si and Al powder with a diameter of 1 µm at the peak wavelength of the blackbody radiation at 2500 K (~1 µm, based on the Wien's law[2]) is around 0.2 (Supplementary Fig. 20a), while for Co, Fe, Ni, and Cr, the transmission factor is ~0.6 (Supplementary Fig. 20a). When the particle diameter is increased to 5 µm in Supplementary Fig. 20b, the transmission factor is smaller for the Co, Fe, Ni, Al, Si, and Cr particle (~0.7–0.8).

Supplementary References:

1. Domalski, E. S., Hearing, E. D. " Condensed Phase Heat Capacity Data " in NIST Chemistry WebBook, NIST Standard Reference Database Number 69, Eds. P.J. Linstrom and W.G. Mallard, National Institute of Standards and Technology, Gaithersburg MD, 20899, https://doi.org/10.18434/T4D303, (retrieved August 8, 2022).
2. Jain, S. C. & Goel, T. C. Thermal conductivity of metals at high temperatures by the Jain and Krishnan method I. Nickel. *J. Phys. D: Appl. Phys.* **1**, 573–580 (1968).
3. Jain, S. C., Narayan, V. & Goel, T. C. Thermal conductivity of metals at high temperatures by the Jain and Krishnan method II. Cobalt. *J. Phys. D: Appl. Phys.* **2**, 101–107 (1969).
4. Touloukian, Y. S., Powell, R. W., Ho, C. Y. & Klemens, P. G. Thermophysical properties of matter - the TPRC data series. Volume 2. Thermal conductivity - nonmetallic solids. (Reannouncement). *Data book*. (U.S. Department of Energy, Office of Scientific and Technical Information, 1971).
5. Powell, R. W., Ho, C. Y. & Liley, P. E. Thermal Conductivity of Selected Materials (U.S. Department of Commerce, National Bureau of Standards, 1966).
6. Coblentz, W. W. The Reflecting Power of Various Metals. (U.S. Government Printing Office, 1911).
7. Zoltán, S., Wolfgang, K., Andreas, K. & Konrad, W. Evaluation of reflectivity of metal parts by a thermo-camera. *InfraMation*, 475-485 (2010).
8. Modest, M. F. & Mazumder, S. Radiative Heat Transfer. (Academic Press, 2021).
9. Palik, E. D. Handbook of Optical Constants of Solids. (Academic Press, 1998).

10. Ordal, M. A., Bell, R. J., Alexander, R. W., Newquist, L. A. & Querry, M. R. Optical properties of Al, Fe, Ti, Ta, W, and Mo at submillimeter wavelengths. *Appl. Opt., AO* **27**, 1203–1209 (1988).

11. Ordal, M. A., Bell, R. J., Alexander, R. W., Long, L. L. & Querry, M. R. Optical properties of Au, Ni, and Pb at submillimeter wavelengths. *Appl. Opt., AO* **26**, 744–752 (1987).

12. Shkondin, E. *et al.* Large-scale high aspect ratio Al-doped ZnO nanopillars arrays as anisotropic metamaterials. *Opt. Mater. Express, OME* **7**, 1606–1627 (2017).

13. Rakić, A. D., Djurišić, A. B., Elazar, J. M. & Majewski, M. L. Optical properties of metallic films for vertical-cavity optoelectronic devices. *Appl. Opt., AO* **37**, 5271–5283 (1998).

14. Zhao, X. *et al.* Optically-switchable thermally-insulating VO2-aerogel hybrid film for window retrofits. *Applied Energy* **278**, 115663 (2020).
